# Supplementary material for: Machine Learning for Human Activity Recognition: State-of-the-Art Techniques and Emerging Trends
Source: J Imaging. 2025 Mar 20;11(3):91. doi: 10.3390/jimaging11030091 (PMC11943402; doi:10.3390/jimaging11030091)
Supplement: Supplementary file 1 [file jimaging-11-00091-s001.zip › jimaging-3510816-supplementary.pdf]

Journal of
Imaging

Supplementary Material S1

Machine Learning for Human Activity Recognition: State-of-the-Art Techniques and Emerging Trends

Md Amran Hossen [1] and Pg Emeroylariffion Abas [1,*]

[1] Faculty of Integrated Technologies, Universiti Brunei Darussalam
* emeroylariffion.abas@ubd.edu.bn

Abstract: Human Activity Recognition (HAR) has emerged as a transformative field with widespread applications, leveraging diverse sensor modalities to accurately identify and classify human activities. This paper provides a comprehensive review of HAR techniques, focusing on the integration of sensor-based, vision-based, and hybrid methodologies. It explores the strengths and limitations of commonly used modalities, such as RGB images/videos, depth sensors, motion capture systems, wearable devices, and emerging technologies like radar and Wi-Fi channel state information. The review also discusses traditional machine learning approaches, including supervised and unsupervised learning, alongside cutting-edge advancements in deep learning, such as convolutional and recurrent neural networks, attention mechanisms, and reinforcement learning frameworks. Despite significant progress, HAR still faces critical challenges, including handling environmental variability, ensuring model interpretability, and achieving high recognition accuracy in complex, real-world scenarios. Future research directions emphasize the need for improved multi-modal sensor fusion, adaptive and personalized models, and the integration of edge computing for real-time analysis. Additionally, addressing ethical considerations, such as privacy and algorithmic fairness, remains a priority as HAR systems become more pervasive. This study highlights the evolving landscape of HAR and outlines strategies for future advancements that can enhance the reliability and applicability of HAR technologies in diverse domains.

Keywords: activity discovery; activity recognition; Sensor Modalities, Machine Learning, Deep Learning, Data Fusion, Emerging Applications

Citation: To be added by editorial staff during production.

Academic Editor: Firstname Lastname

Received: date
Revised: date
Accepted: date
Published: date

Copyright: © 2024 by the authors. Submitted for possible open access publication under the terms and conditions of the Creative Commons Attribution (CC BY) license (https://creativecommons.org/licenses/by/4.0/).

1. Introduction

This Supplementary Material S1 provides a comprehensive tabulation of publicly available datasets and methods for Human Activity Recognition (HAR), categorized by data modality and methodology. These tables are supplementary to the manuscript titled "Machine Learning for Human Activity Recognition: State-of-the-Art Techniques and Emerging Trends", offering additional reference material to support the discussions presented in the paper.

While essential for completeness, these tables have been moved from the main manuscript to ensure a more streamlined discussion. The tables provide insights into key dataset characteristics, including the number of action samples, subjects, viewpoints, and year of publication. Additionally, various machine learning and deep learning-based HAR methods are summarized for reference.

The datasets and methods are organized as follows:

- Table S1: Vision-based HAR datasets
- Table S2: Depth and skeleton-based HAR datasets
- Table S3: Wearable and mobile accelerometer-based HAR datasets
- Table S4: Motion capture (MoCap)-based HAR datasets

- Table S5: Wi-Fi and radar-based HAR datasets
- Table S6: Multimodal HAR datasets
- Table S7: Other sensor modalities used in HAR

Additionally, the following tables summarize machine learning and deep learning-based HAR methods:

- Table S8: Machine learning-based HAR using different modalities
- Table S9: Unsupervised learning-based HAR methods
- Table S10: Semi-supervised learning-based HAR methods
- Table S11: Deep learning-based HAR methods
- Table S12: Transformer-based HAR methods
- Table S13: Reinforcement learning-based HAR methods

These tables serve as a valuable resource for researchers seeking an overview of available datasets and methodological advancements in HAR, complementing the discussions presented in the main paper.

2. Tabulations on different Datasets and Methods in HAR

Tables S1-S7 summarize the key publicly available datasets for HAR, organized by modality, including vision-based, depth and skeleton-based, wearable and mobile accelerometer-based, motion capture (mocap), Wi-Fi and radar-based, multimodal, and other sensor types. In these tables, the sources listed in the first column represent the original works that introduced the respective datasets. For each dataset, key characteristics such as the number of action samples, number of views, number of human subjects, and the year of publication are highlighted, providing a detailed overview of available datasets for research. Each dataset modality provides unique advantages and challenges, making them suitable for different use cases and research purposes. The tabular organization facilitates a clearer understanding of their relevance for HAR research and development.

Table S1: Summary of publicly available Publicly available vision-based dataset for HAR.

Dataset	Year	Modality	#Class	#Subject	#Sample	#View-point
UCF activity data set [1]	2001	RGB				
KTH [2]	2004	RGB	6	25	2,391	1
Weizmann [3]	2005	RGB	10	9	90	1
TSA airport tarmac dataset [4]	2005	RGB				
INRIA data set [5]	2006	RGB				
IXMAS [5]	2006	RGB	11	10	330	5
HDM05 [6]	2007	RGB	130	5	2,337	1
Hollywood [7]	2008	RGB	8	-	430	-
Hollywood2 [8]	2009	RGB	12	-	3,669	-
MSR-Action3D [9]	2010	RGB,S,D	20	10	567	1
Olympic [10]	2010	RGB	16	-	783	-
CAD-60 [11]	2011	RGB,S,D	12	4	60	-
HMDB51 [12]	2011	RGB	51	-	6,766	-
RGB-HuDaAct [13]	2011	RGB,D	13	30	1,189	1
ACT42 [14]	2012	RGB,D	14	24	6,844	4
DHA [15]	2012	RGB,D	17	21	357	1
MSRDailyActivity3D [16]	2012	RGB,S,D	16	10	320	1
UCF101 [17]	2012	RGB	101	-	13,320	-

CAD-120 [18]	2013	RGB,S,D	10	4	120	-
IAS-lab [19]	2013	RGB,S,D,Ps	15	12	540	1
J-HMDB [20]	2013	RGB,S	21	-	31,838	-
MSRAction-Pair [21]	2013	RGB,S,D	12	10	360	1
Multi-View TJU [22]	2014	RGB,S,D	20	22	7,040	2
Northwestern-UCLA [23]	2014	RGB,S,D	10	10	1,475	3
Sports-1M [24]	2014	RGB	487	-	1,113,158	-
UWA3D Multiview [25], [26]	2014	RGB,S,D	30	10	~900	4
ActivityNet [27]	2015	RGB	203	-	27,801	-
SYSU 2D HOI [28]	2015	RGB,S,D	12	40	480	1
THUMOS Challenge 15 [29]	2015	RGB	101	-	24,017	-
TJU [30]	2015	RGB,S,D	15	20	1,200	1
UTD-MHAD [31]	2015	RGB,S,D,Ac,Gyr	27	8	861	1
UWA3D Multiview II [32]	2015	RGB,S,D	30	10	1,075	
Charades [33]	2016	RGB	157	267	9,848	-
InfAR [34]	2016	IR	12	40	600	2
PiGR [35]	2016	RGB, S				
NTU RGB+D RGB+D [36]	2016	RGB,S,D,IR	60	40	56,880	80
AVA [37]	2017	RGB	80	-	437	-
DvsGesture [38]	2017	Event Stream	17	29	-	-
FCVID [39]	2017	RGB	239	-	91,233	-
Kinetics-400 [40]	2017	RGB	400	-	306,245	-
NEU-UB [41]	2017	RGB,D	6	20	600	-
PKU-MMD [42]	2017	RGB,S,D,IR	51	66	1,076	3
Something-Something-v1 [43]	2017	RGB	174	-	108,499	-
MPI-INF-3DHP [44]	2017	RGB, D, S				
EPIC-KITCHENS-55 [45]	2018	RGB, Au	-	32	39,594	Egocentric
Kinetics-600 [46]	2018	RGB	600	-	495,547	-
RGB-D Varying-view [47]	2018	RGB,S,D	40	118	25,600	8+1(360◦)
DHP19 [48]	2019	ES,	S	33	17	-
Drive&Act [49]	2019	RGB,S,D,IR	83	15	-	6
Egogesture [50]	2018	RGB, D	83	50	2,953,224	
Moments in time [51]	2019	RGB	339	-	~1,000,000	-
NTU RGB+D 120 [52]	2019	RGB,S,D,IR	120	106	114,480	155
PROX [53]	2019	RGB, S				
ETRI-Activity3D [54]	2020	RGB,S,D	55	100	112,620	-
EV-Action [55]	2020	RGB,S,D,EMG	20	70	7,000	9
FlickrCI3D [56]	2020	RGB, S		81,302	11,770	
GRAB [57]	2020	Body Mesh	4	10		
IKEA ASM [58]	2020	RGB,S,D	33	48	16,764	3
RareAct [59]	2020	RGB	122	-	905	-
UBD-kinect dataset [60]	2020	RGB, D, S	17	3	115,270	3
MoVi [61]	2021	RGB, Mocap	21	90	7,344,000+	4
UAV-Human [62]	2021	RGB,S,D,IR,etc.	155	119	67,428	-

HOMAGE [63]	2021	RGB, IR, Au,Ac, Gy,	86		26,000+	5
Ego4D [64]	2022	RGB,Au,Ac,etc.	-	923	-	Egocentric
EPIC-KITCHENS-100 [65]	2022	RGB,Au,Ac	-	45	89,979	Egocentric
EPIC-KITCHENS-55 [45]	2023	RGB	-	397	2,000,000+	Egocentric

Table S2: Publicly available Depth and skeleton-based dataset for HAR.

Dataset	Year	Modality	#Class	#Subject	#Sample	#View-point
MSR-Action3D [9]	2010	RGB,S,D	20	10	567	1
CAD-60 [11]	2011	RGB,S,D	12	4	60	-
RGB-HuDaAct [13]	2011	RGB,S,D	13	30	1,189	1
ACT42 [14]	2012	RGB,S,D	14	24	6,844	4
DHA [15]	2012	RGB,S,D	17	21	357	1
UCFKinect [66]	2013	S	16	16	1,280	1
MSRAction-Pair [21]	2013	RGB,S,D	12	10	360	1
Multi-View TJU [22]	2014	RGB,S,D	20	22	7,040	2
Northwestern-UCLA [23]	2014	RGB,S,D	10	10	1,475	3
UPCV [67]	2014	S	10	20	400	1

Table S3: Publicly available wearable and mobile accelerometer-based dataset for HAR.

Dataset	Year	Modality	#Class	#Subject	#Sample	#Sensors
WISDM [68]	2011	Ac	6	29	1,098,207	-
SCAR [69]	2012	AC,Gy	8	10		3
PAMAP2 [70]	2012	Ac, Gy	18	9	-	-
USC-HAD [71]	2012	AC	12	-	-	-
OPPORTUNITY [72]	2013	Ac	35	-	-	-
ADL [73]	2013	Ac	14	30	192	-
OPPORTUNITY	2013	Ac	18	12		
UniMiB SHAR [74]	2017	Ac	17	30	11,771	-
W-HAR[75]	2020	Ac	8	22		2
HARTH [76]	2021	Ac	12	22		2
DanHAR [77]	2021	Ac/Mobile	5	10		3
KU-HAR[78]	2021	Ac/Gy/Mob	18	90	20750	
Daily living activities [79]	2021	Ac	17	8		

Table S4: Publicly available Mocap datasets for HAR and motion analysis.

Dataset	Year	Modality	#Class	#Subject	#Sample	#View-point
HUMANEVA [80]	2006	Mocap, S	4		40,000	4
CMU Mocap Dataset [81]	2007	Mocap, S	30	140	500,000+	
Human3. 6m [82]	2013	RGB, D, Mocap		11	1.46M+	4
MPI-INF-3DHP [44]	2017	Mocap	8	8	1.3M+	14
3DPW [83]	2018	Mocap, RGB	-	5	51, 000	Multiview
LARa [84]	2020	Mocap	8	474		
BABEL [85]	2021	Mocap	252	-	13,220	-

HumanSC3D [86]	2021	RGB, S, Mocap	172	6	3.1M+	2
Fit3D [87]	2021	RGB, S, Mocap		37	2.9M+	5
MoVi [61]	2021	RGB, Mocap	21	90	7.3M+	4
MMVP [88]	2024	Mocap	10	16	44000+	

Table S5: Publicly available Wi-Fi and Radar signature-based datasets for HAR.

Dataset	Year	Modality	#Class	#Subject	#Sample	#Sensors
SignFi [89]	2018	Wifi	276	11		
Hernang ́omez et al [90]	2019	Radar	8	11	1,056	
Wang et al. [91]	2019	WiFi	CSI	6	1	2
UG-Radar [92]	2019	Radar	6	5		
Widar 3.0 [93]	2019	Wifi	6	-	258000+	-
UT-HAR [94]	2019	Wifi	6	6	5000	
DIAT-MRADHAR [95]	2022	Radar	6		3780	
EfficientFi [96]	2022	Wifi	6	8	12000	
NTU-HumanID [97]	2022	Wifi	14	14	850+	

Table S6: Multimodal datasets Publicly available for HAR.

Dataset	Year	Modality	#Class	#Subject	#Sample	#View-point
MSR-Action3D [9]	2010	RGB,S,D	20	10	567	1
Berkeley MHAD [98]	2013	RGB,S,D,Au,Ac	12	12	660	4
CAD-120 [18]	2013	RGB,S,D	10	4	120	-
IAS-lab [19]	2013	RGB,S,D,PC	15	12	540	1
J-HMDB [20]	2013	RGB,S	21	-	31,838	-
MSRAction-Pair [21]	2013	RGB,S,D	12	10	360	1
UTD-MHAD [31]	2015	RGB,S,D,Ac,Gy	27	8	861	1
NTU RGB+D RGB+D [36]	2016	RGB,S,D,IR	60	40	56,880	80
PKU-MMD [42]	2017	RGB,S,D,IR	51	66	1,076	3
MADS [99]	2019	RGB, D, S, Mocap	30	5	53,000	
MMAct [100]	2019	RGB,S,Ac,Gyr	37	20	36,764	4+Egocentric
Drive&Act [49]	2019	RGB,S,D,IR	83	15	-	6
HOMAGE [63]	2021	RGB,IR,Ac,Gy	75	27	1,752	2~5
FlickrSC3D self-contact [86]	2021	RGB, S				
UAV-Human [62]	2021	RGB,S,D,IR,etc.	155	119	67,428	-
HOMAGE [63]	2021	RGB, IR, Au,Ac, Gy	86		26,000+	5
Ego4D [64]	2022	RGB,Au,Ac,etc.	-	923	-	Egocentric
EPIC-KITCHENS-100 [65]	2022	RGB,Au,Ac	-	45	89,979	Egocentric

* Gy= Gyroscope, S = Skeleton, D = Depth, Ir = Infra-Red, Au = Audio, Ac = Acceleration, Ps = Pressure.

Table S7: Other Data Modalities used by HAR researchers

Dataset	Year	Modality	#Class	#Subject	#Sample	#View-point
Berkeley MHAD [98]	2013	Audio	12	12	660	4
DVS128 Gesture [38]	2017	Event stream	11	29		3
Bathroom Activities [101]	2020	Proximity sensors	3	-	-	-
Ego4D [64]	2022	Audio	-	923	-	-
LAMAR[102]	2021	LIDAR	7	7	-	-
OPERAnet [103]	2022	Radio-Frequency	8	-	-	-

3. Tabulations of Machine Learning and Deep Learning-Based HAR Methods

Tables S8-S13 provide a comprehensive overview of various HAR methods from the literature. In these tables, the methods listed in the first column represent the original works that used the respective datasets in the last column. Additionally, the year of publication, data modality and top accuracy are highlighted by providing a detailed overview of the method. Table S8 focuses on classical machine learning techniques, while Table S9 highlights unsupervised learning methods. Semi-supervised learning strategies are detailed in Table S10, and Table S11 lists various deep learning-based approaches. Table S12 examines the use of transformers for HAR, and Table S13 explores reinforcement learning methods.

Table S8: Machine learning based HAR using different modalities

Method	Year	Modality	Top Accuracy %	Dataset
HAR based on Decision Tree (DT) [104]	2013	Mobile Phone	88.32	[104]
HAR based on Decision Tree (DT) using acceleration and Jerk [105]	2021	Mobile Phone Acc + Jerk	89.32	[105]
HAR based on DT using data from wearable [106]	2021	Wearable Acc	99.1, 89.5	[106]
Exercise quality monitoring [107]	2021	Wearable Acc	85.5	[107]
Real-Time Activity Recognition Using PDA and DT [108]	2010	PDA and WMB	94.0	[108]
HAR using PCA and random forest (RF)[109]	2018	Smart Watch	99	[109]
HAR based on evolution of features selection and RF [110]	2019	Wearable	96	[110]
HAR based on spatiotemporal interest point (STIP) [111]	2014	3D depth map	94.3, 91.0, 87.5	MSR, UTK, CAD-60
HAR using ax-min features and key poses [112]	2017	Skeleton	81.83	CAD-60
Daily activity recognition using RF [113]	2020	Smartphone	84.78	HCI-HAR [73]
HAR using APJ3D and RF [114]	2013	Skeleton	92	UTK
HAR based on key poses and SVM [115]	2016	Skeleton	95.0	KARD
HAR in in smartphone using support vector machine [116]	2016	Smartphone	89.59	[116]
HAR using multiclass hardware friendly SVM [117]	2016	Smartphone	89.0	[117]

HAR based on Micro-Doppler Signatures and SVM [118]	2009	Radar	90	[118]
Hybrid HAR using SVM and 1D CNN [119]	2020	Wearable	97.71	UCI-HAR
HAR using MC-SVM-Linear[120]	2017	Smartphone	98.57	UCI-HAR
K-Nearest Neighbors (k-NN) [121]	2021	Smartphone	96.5	UCI-HAR
P-HAR using wearable sensors [122]	2015	Wearable	96.53	[122]
Har using Smartphone and K-NN [123]	2012	Smartphone	-	[123]
RGBD HAR using multi-Features Combination and k-NN [124]	2017	RGBD image	85.71%	MSR
HAR using smartphone and k-NN [125]	2018	Smartphone	97.9	[125]
Har using M-LR [126]	2015	Vibration	74	[126]
HAR using hidden process regression [127]	2013	Wearable	90.3	[127]
Comparative analysis of HAR based on LR [128]	2020	Smartphone	96.1, 94.5	UCI HAR, HAPT
Performance comparison activity recognition using LR and SVM [129]	2020	Smartphone	96.19, 94.02	UCI HAR,
Evaluation of HAR using Smartphone [130]	2021	Smartphone	98.1, 97, 91	Pamap2, mHealth and SWELL
FedHAR [131], SGD	2018	Smartphone	93.01	UCI
Evaluation of HAR [132]	2020	Smartphone	96.02	UCI
S-HAR [133]	2022	Wearable	91.08, 91.45, 93.16	USC-HAD, IM-WSHA, and MO-TIONSENE
Automatic Har using CNN [134]	2014	Wearable	90.3, 76.2, 96.5	Skoda, Opportunity, Actitracker
HAR using fast stochastic gradient descent (FSGD) [135]	2016	Wearable	93.2	[135]
HAR based on NB using data from wearable	2021	Wearable	89.5	[106]
Naïve Bayes [136]	2020	Smart home	89.5	[136]
Online-Har using NB [137]	2012	Smartphone	91.1	[137]
HAR using Smooth NB classifier [138]	2010	Smartphone	83	[138]
Smartphone based HAR using XGBoost[139]	2019	Smartphone	84.41	[139]
HAR based on MOT using XGBoost [140]	2024	RGB image	91	SBU
Evaluation of Deep HAR [141]	2019	Wearable	89.97	MHEALTH
Pose-based HAR [142]	2016	Skeleton	99.82, 96.64	MSR, UTK
Evaluation of ML based HAR including XGBoost [130]	2021	Wearable	99.1,97.1, 93.2	Pamap2, Mhealth, SWELL
Evaluation of ML based HAR including NGBoost [130]	2021	Wearable	93.6,88.1, 87.5	Pamap2, Mhealth, SWELL
HAR with HMM-DNN [143]	2015	Smartphone	93.5	[143]
HAR using TSC-HMM [144]	2014	Smartphone	91.76	UCI
HAR using MAP-HMM [145]	2016	Smartphone	93.3	[145]

HOJ3D based HAR using HMM [146]	2012	Skeleton	95.0	[146]

* Table keys, PDA = Personal Digital Assistant, WMB = wireless motion bands, STIP = spatiotemporal interest point, MC = Multi Class, MOT = Multiple object tracking,

Table S9: Unsupervised learning based HAR using different modalities

Method	Year	Modality	Top accuracy %	Datasets
HAR using K-means clustering [122]	2015	Wearable	83.1	[122]
Unsupervised feature learning for HAR [147]	2014	Smartphone	92.16	UCI HAR
Unsupervised HAR [60]	2022	Skeletons	58.02, 68.1, 61.3	UTK, CAD-60, UBD-Kinect
HAR based on parallel approximation kernel k-means [148]	2020	Smartphone	61.2	UCI HAR
HAR using dynamic k-means [149]	2017	Skeletons	95.3, 92.7	CAD-60, TST
Comprehensive review of unsupervised HAR [150]	2020	Wearable	-	-
HAR using Spectral clustering [60]	2022	Skeletons	55.02, 63.2, 60.1	UTK, CAD-60, UBD-Kinect
HAR based on 3D-pose and clustering [151]	2015	Skeletons	77.3, 76.7	KARD, CAD-60
HAD using sensor [152]	2015	Smartphone	88.57	UCI
HAR using agglomerative clustering	2020	Skeletons	52.05, 64, 60.1	UTK, CAD-60, UBD-Kinect
HAR using agglomerative clustering [147]	2014	Smartphone	74.1	[147]
B-DSAFEC [153]	2021	RGB	91	UCF
Hierarchical clustering multi-task learning or joint human action grouping and recognition [154]	2016	RGB	78.5, 95.0, 89.7, 93.2, 51.4, 76.3	Hollywood2, UCF Sports, YouTube, UCF50, HMDB51, UCF101
ClusterFL for HAR on edge device [155]	2022	RGB	71.20	[155]
HAR using DBSCAN clustering [147]	2014	Smartphone	90.1	[147]
HAR based on Action primitives [156]	2017	Wearable	55.1	[156]
Unsupervised HAD using DBSCAN [157]	2018	Wearable	56.3	HHAR
HAR based on parallel approximation DBSCAN [148]	2020	Smartphone	52.1	UCI HAR
HAR in smart home using clustering [158]	2014	Smart home	91.40, 94.71, 93.36	Aruba, Kasteren7, Kasteren10
Gaussian mixture based HMM for D-HAR using 3D skeleton features [159]	2013	Skeleon	84.02	CAD-60
Continuous Activity Recognition Using GMM–HMM [160]	2022	Wi-Fi	72.1	[160]
Hierarchical Human Activity Recognition Using GMM [161]	2012	Wearable	86.9	WARD

Table S10: Semi supervised HAR methods from the literature.

Method	Year	Modality	Top Accuracy	Dataset
FedHAR [162]	2021	Smartphone	92.2, 85.5	MobiAct [163], WISDM [164]
Semi-Supervised Adversarial Learning Using LSTM [165]	2022	Smart Home	95.0, 96.2	Kasteren [166], CASAS [167]

SATL [168]	2021	Wearable	95,01, 97.1	HCI-HAR, mHealth
Context-Aware Mutual Learning [169]	2023	Wearable	71.1, 79.0, 65.2, 65.09	HCI-HAR [73], WISDM, PAMAP2 [170], mHealth [171]
Multi-modal DEC [172]	2020	multi-modal	66.56	Instagram Dataset [172]
Active Semi-Supervised Deep Learning [173]	2023	Wearable	76.1, 45.0, 91.02	PAMAP2, USC-HAD and UCI-HAR
Semi-Supervised Ensemble Learning [174]	2024	Wearable	75.01	CASAS
Federated Clustering and Semi-Supervised Learning [175]	2023	Wearable	79.5, 83.05	HCI-HAR [250], WISDM,
Semi-Supervised Convolutional Neural Networks [176]	2018	Wearable	65.5, 58.1, 71.1	ActiTracker [68], PAMAP2, mHealth
Self-Supervised Learning with Generative Adversarial Networks [177]	2023	Multimodal	84.7, 71.05, 72.01	PAMAP2, opportunity-locomotion, LISSI

Table S11: Various deep learning-based methods published in recent years.

Method	Year	Modality	Top accuracy	Dataset
Scene Flow to Action Map (SFAM) with CNN [178]	2017	RGBD	36.27, 89.4	ChaLearn LAP RGB-D [179], F2I [180]
CNN [181]	2017	Skeletons	93.47, 75.94, 89.46	SBU Kinect, NTU RGB+D, CMU
CNN [182]	2017	Skeletons	75.2, 84.2	NTU RGB+D, UWA3DII
CNN with Transfer learning [44]	2017	2D and 3D skeletons	73.5	
CNN with event stream [48]	2019	Event stream	79.6	
ST-GCN [183]	2018	RGB-D, Skeletons	88.3, 73.2, 30.7, 25.2	NTU60 , NTU120, Kinetics , FineGYM
TSN [184]	2018	RGB-D, Skeletons		
AS-GCN [185]	2020	RGB-D, Skeletons	94.19, 79.8, 34.8	NTU60 , NTU120, Kinetics
RA-GCN [186]	2020	RGB-D, Skeletons	93.6, 82.7,	NTU60 , NTU120,
2S-AGCN [187]	2019	RGB	95.1	Kinetics
DGNN [188]	2019	RGB	96.1%	
FGCN [189]	2021	RGB	96.3, 87.4	NTU60 , NTU120,
Shift-GCN [190]	2020	RGB	96.5, 85.9, 94.6	NTU60, NTU120, NWCLA
DSTA-Net [191]	2020	RGB	91.5, 96.4, 86.4, 89.0	SHREC, DHG, NTU60 , NTU120,
MS-G3D [192]	2020	Skeletons	96.2, 88.4, 60.9	NTU60 , NTU120, Kinetics Skeleton 400
STSS [193]	2021	Mulit-modal	41.6 , 84.4, 91.1, 87.7	Diving-48, Something-Something-V1 & V2, FineGym
HAC [194]	2020	Mulit-modal R + P	95.8, 93.6, 93.9, 91.6	NTU60,NTU 120, N-UCLA, UTD-MHAD

VPN++ [195]	2021	Mulit-modal R + P	71.0, 90.7, 86.2, 93.5	Smarthome, NTU60,NTU 120, N-UCLA
VS-Transformers [196]	2022	Mulit-modal R	84.9, 85.9, 69.6	Kinetics-400, Kinetics-600, and Something-Something V2
OmniSource [197]	2020	Mulit-modal R + F	96.4, 71.7, and 83.6	UCF101, HMDB51, Kinetics400
PoseConv3D [198]	2022	3D heatmap volume	97.1, 90.3, 47.7, 94.3, 76.7, 55.6	NTU60, NTU120, Kinetics, FineGYM, HMDB51, and UCF101

* Keys R = RGB, P = Pose, S = Skeleton,

Table S12: Transformers for HAR,

Method	Year	Modality	Top Accuracy	Dataset
I3D [199]	2021	RGB, Flow RGB + Flow	97.9, 80.2, 78.7	UCF-101, HMDB, Kinetics
Girdhar et al. [200]	2019	RGB	- mAp was used	K400, AVA
GroupFormer [201]	2021	RGB	95.7, 96.3	Volleyball, Collective
VTN [202]	2021	RGB	78.6, 65.4	K400, MT
VidTr [203]	2021	Pixel Patch	70.2, 43.5, 63.0, 96.7, 74.4	K400, Charades, SSV2, UCF-101, HMDB-51
TimeSformer [204]	2021	RGB	78.0, 59.5	K400, SSV2
X-ViT [205]	2021	RGB	89.3, 93.7, 64.7	SSV2, K400, Epic-K
Mformer [206]	2021	RGB	95.2, 90.6, 58.5	K400, SSV2, Epic-K
MViT [207]	2021	RGB	95.1, 96.5, 91.5, 47.7, 28.7	K400, K600, SSV2, Charade, AVA
ViViT [208]	2021	RGB	75.8, 64.1, 38.8, 65.9, 64.1	K400, K600, EpicK , SSV2, MT,
TokenLearner [209]	2021	Image-Like Tensor	91.05, 85.4, 86.3, 66.3, 53.8	ImageNet, K400, K600, Charades, AViD
SCT [210]	2021	RGB	78.41, 96.3, 98.02, 97.0	K400, K600, UCF-101, HMDB
DETR [211]	2022	RGB	36.08, 61.3	HICO-DET [212], V-COCO [213]
UGPT [214]	2022	RGB	67.82, 81.42, 42.4	Breakfast Actions [215], MultiTHUMOS [216], and Charades.
RegionViT [217]	2022	RGB	77.6, 59.8	K400, SSV2
RViT-XL [218]	2022	RGB	81.5, 92.31, 67.9, 66.1	K400, Jester [219], SSV2, Charades
DirecFormer [220]	2022	RGB	98.15 , 94.86 , 64.94	Jester, K400, SSV2
UniFormer [221]	2022	RGB	82.9, 84.8, 60.9, 71.2	K400, J600, SSV1, SSV2
SIFAR [222]	2022	RGB	87.9, 94.4, 97.2, 87.3	K400, SSV2, Jester, Diving48
MTV [223]	2022	RGB	82.4, 89.6, 82.2, 48.6, 68.5, 47.2	K400, K600, K700, EpicK, SSV2, MT
SVT [224]	2022	RGB	78.1, 93.7, 67.2, 59.2	K400, UCF101, HMDB51, SSV2

BEVT [225]	2022	RGB	80.6, 70.6, 86.7	K400, SSV2, Diving48
VideoMAE [226]	2022	RGB	87.4, 75.4, 91.3, 62.6	K400, SSV2, UCF101, HMDB51
ST-MAE [227]	2022	RGB	84.9, 73.6, 73.6	K400, SSV2, AVA
LSTCL [228]	2022	RGB	76.6, 82.2, 67.7, 75.9, 96.8	K400, K600, SSV2, HMDB, UCF101
OmniMAE [229]	2023	RGB	82.8, 68.6	ImageNet [230], SSV2
MME [231]	2023	RGB	81.8, 70.5, 96.5, 78.0	K400, SSV2, UCF101, HMDB51
MGM [232]	2023	RGB	81.7, 72.1, 97.7, 81.0, 82.6	K400, SSV2, UCF101, HMDB51, Diving48

Dataset keys Kinetics-400 = K400 [199], UCF101 [17], HMDB51 [12], SSV2 = Something-Something V2 [43], K = Kinetics, MT = Moments in Time, SSV2 = Something something V2, Epic-K = Epic Kitchens, AVA = Atomic Visual Actions

Table S13: Reinforcement learning based HAR

Method	Year	Modality	Top Accuracy	Dataset
Spatio-Temporal Deep Q-Network for HAR localization [233]	2021	RGB	0.78, 0.79, 45.85, 0.67	UCF-Sports, UCF-101, ActivityNet, JHMDB,
Deep RL for skeleton based HAR [234]	2021	RGB, Skeleton	79.6, 98.5, 89.8	SYSU-3D [207], UT-Kinect [86], NTU 60
Deep Q-Learning for video-Based HAR [235]	2021	RGB	85.70	ActivityNet [27]
Actor-Critic Model for Activity Recognition [236]	2019		-	DoM-SEV
HAR from mobile robot platform with DRL [237]	2019	RGB (Virtual)	75.10	HoME
Location independent HAR using wifi and DRL [238]	2021	Wifi	80.05, 83.01	Yousefi-2017 [94] , Fall-DeFi [239]
Deep Q-Network for Feature Selection [240]	2021	RGB	68.4, 44.9, 55.9	UCF101 [17], HMDB51 [12], Olympic Sports [10]

References

1. Rao, C.; Shah, M. View-Invariance in Action Recognition. In Proceedings of the Proceedings of the 2001 IEEE Computer Society Conference on Computer Vision and Pattern Recognition. CVPR 2001; 2001; Vol. 2, pp. II--II.

2. Schuldt, C.; Laptev, I.; Caputo, B. Recognizing Human Actions: A Local SVM Approach. In Proceedings of the Proceedings of the 17th International Conference on Pattern Recognition, 2004. ICPR 2004.; 2004; Vol. 3, pp. 32-36 Vol.3.

3. Gorelick, L.; Blank, M.; Shechtman, E.; Irani, M.; Basri, R. Actions as Space-Time Shapes. *IEEE Trans. Pattern Anal. Mach. Intell.* **2007**, *29*, 2247–2253.

4. Vaswani, N.; Roy-Chowdhury, A.K.; Chellappa, R. " Shape Activity": A Continuous-State HMM for Moving/Deforming Shapes with Application to Abnormal Activity Detection. *IEEE Trans. Image Process.* **2005**, *14*, 1603–1616.

5. Weinland, D.; Ronfard, R.; Boyer, E. Free Viewpoint Action Recognition Using Motion History Volumes. *Comput. Vis. image Underst.* **2006**, *104*, 249–257.

6. Müller, M.; Röder, T.; Clausen, M.; Eberhardt, B.; Krüger, B.; Weber, A. Mocap Database Hdm05. *Inst. für Inform. II, Univ. Bonn* **2007**, *2*.

7. Laptev, I.; Marszalek, M.; Schmid, C.; Rozenfeld, B. Learning Realistic Human Actions from Movies. In Proceedings of the 2008 IEEE conference on computer vision and pattern recognition; 2008; pp. 1–8.

8. Marszalek, M.; Laptev, I.; Schmid, C. Actions in Context. In Proceedings of the 2009 IEEE Conference on Computer Vision and Pattern Recognition; 2009; pp. 2929–2936.

9. Li, W.; Zhang, Z.; Liu, Z. Action Recognition Based on a Bag of 3d Points. In Proceedings of the 2010 IEEE computer society conference on computer vision and pattern recognition-workshops; 2010; pp. 9–14.

10. Niebles, J.C.; Chen, C.-W.; Fei-Fei, L. Modeling Temporal Structure of Decomposable Motion Segments for Activity Classification. In Proceedings of the Computer Vision--ECCV 2010: 11th European Conference on Computer Vision, Heraklion, Crete, Greece, September 5-11, 2010, Proceedings, Part II 11; 2010; pp. 392–405.

11. Sung, J.; Ponce, C.; Selman, B.; Saxena, A. Human Activity Detection from RGBD Images. In Proceedings of the Proceedings of the 16th AAAI Conference on Plan, Activity, and Intent Recognition; AAAI Press, 2011; pp. 47–55.

12. Kuehne, H.; Jhuang, H.; Garrote, E.; Poggio, T.; Serre, T. HMDB: A Large Video Database for Human Motion Recognition. In Proceedings of the 2011 International conference on computer vision; 2011; pp. 2556–2563.

13. Ni, B.; Wang, G.; Moulin, P. Rgbd-Hudaact: A Color-Depth Video Database for Human Daily Activity Recognition. In Proceedings of the 2011 IEEE international conference on computer vision workshops (ICCV workshops); 2011; pp. 1147–1153.

14. Cheng, Z.; Qin, L.; Ye, Y.; Huang, Q.; Tian, Q. Human Daily Action Analysis with Multi-View and Color-Depth Data. In Proceedings of the Computer Vision--ECCV 2012. Workshops and Demonstrations: Florence, Italy, October 7-13, 2012, Proceedings, Part II 12; 2012; pp. 52–61.

15. Lin, Y.-C.; Hu, M.-C.; Cheng, W.-H.; Hsieh, Y.-H.; Chen, H.-M. Human Action Recognition and Retrieval Using Sole Depth Information. In Proceedings of the Proceedings of the 20th ACM international conference on Multimedia; 2012; pp. 1053–1056.

16. Wu, Y. *Mining Actionlet Ensemble for Action Recognition with Depth Cameras*; 2012;

17. Soomro, K.; Zamir, A.R.; Shah, M. UCF101: A Dataset of 101 Human Actions Classes From Videos in The Wild. **2012**.

18. Koppula, H.S.; Gupta, R.; Saxena, A. Learning Human Activities and Object Affordances from RGB-D Videos. *Int. J. Rob. Res.* **2013**, *32*, 951–970, doi:10.1177/0278364913478446.

19. Munaro, M.; Ballin, G.; Michieletto, S.; Menegatti, E. 3D Flow Estimation for Human Action Recognition from Colored Point Clouds. *Biol. Inspired Cogn. Archit.* **2013**, *5*, 42–51.

20. Jhuang, H.; Gall, J.; Zuffi, S.; Schmid, C.; Black, M.J. Towards Understanding Action Recognition. In Proceedings of the Proceedings of the IEEE international conference on computer vision; 2013; pp. 3192–3199.

21. Oreifej, O.; Liu, Z. Hon4d: Histogram of Oriented 4d Normals for Activity Recognition from Depth Sequences. In Proceedings of the Proceedings of the IEEE conference on computer vision and pattern recognition; 2013; pp. 716–723.

22. Liu, A.-A.; Su, Y.-T.; Jia, P.-P.; Gao, Z.; Hao, T.; Yang, Z.-X. Multiple/Single-View Human Action Recognition via Part-Induced Multitask Structural Learning. *IEEE Trans. Cybern.* **2014**, *45*, 1194–1208.

23. Wang, J.; Nie, X.; Xia, Y.; Wu, Y.; Zhu, S.-C. Cross-View Action Modeling, Learning and Recognition. In Proceedings of the Proceedings of the IEEE conference on computer vision and pattern recognition; 2014; pp. 2649–2656.

24. Karpathy, A.; Toderici, G.; Shetty, S.; Leung, T.; Sukthankar, R.; Fei-Fei, L. Large-Scale Video Classification with Convolutional Neural Networks. In Proceedings of the Proceedings of the IEEE conference on Computer Vision and Pattern Recognition; 2014; pp. 1725–1732.

25. Rahmani, H.; Mahmood, A.; Q Huynh, D.; Mian, A. HOPC: Histogram of Oriented Principal Components of 3D Pointclouds for Action Recognition. In Proceedings of the Computer Vision--ECCV 2014: 13th European Conference, Zurich, Switzerland, September 6-12, 2014, Proceedings, Part II 13; 2014; pp. 742–757.

26. Rahmani, H.; Mahmood, A.; Huynh, D.; Mian, A. Action Classification with Locality-Constrained Linear Coding. In Proceedings of the 2014 22nd International Conference on Pattern Recognition; 2014; pp. 3511–3516.

27. Caba Heilbron, F.; Escorcia, V.; Ghanem, B.; Carlos Niebles, J. Activitynet: A Large-Scale Video Benchmark for Human Activity Understanding. In Proceedings of the Proceedings of the ieee conference on computer vision and pattern recognition; 2015; pp. 961–970.

28. Hu, J.-F.; Zheng, W.-S.; Lai, J.; Zhang, J. Jointly Learning Heterogeneous Features for RGB-D Activity Recognition. In Proceedings of the Proceedings of the IEEE conference on computer vision and pattern recognition; 2015; pp. 5344–5352.

29. Gorban, A.; Idrees, H.; Jiang, Y.-G.; Zamir, A.R.; Laptev, I.; Shah, M.; Sukthankar, R. THUMOS Challenge: Action Recognition with a Large Number of Classes 2015.

30. Liu, A.-A.; Nie, W.-Z.; Su, Y.-T.; Ma, L.; Hao, T.; Yang, Z.-X. Coupled Hidden Conditional Random Fields for RGB-D Human Action Recognition. *Signal Processing* **2015**, *112*, 74–82.

31. Chen, C.; Jafari, R.; Kehtarnavaz, N. UTD-MHAD: A Multimodal Dataset for Human Action Recognition Utilizing a Depth Camera and a Wearable Inertial Sensor. In Proceedings of the 2015 IEEE International conference on image processing (ICIP); 2015; pp. 168–172.

32. Rahmani, H.; Mahmood, A.; Huynh, D.; Mian, A. Histogram of Oriented Principal Components for Cross-View Action Recognition. *IEEE Trans. Pattern Anal. Mach. Intell.* **2016**, *38*, 2430–2443.

33. Sigurdsson, G.A.; Varol, G.; Wang, X.; Farhadi, A.; Laptev, I.; Gupta, A. Hollywood in Homes: Crowdsourcing Data Collection for Activity Understanding. In Proceedings of the Computer Vision--ECCV 2016: 14th European Conference, Amsterdam, The Netherlands, October 11--14, 2016, Proceedings, Part I 14; 2016; pp. 510–526.

34. Gao, C.; Du, Y.; Liu, J.; Lv, J.; Yang, L.; Meng, D.; Hauptmann, A.G. Infar Dataset: Infrared Action Recognition at Different Times. *Neurocomputing* **2016**, *212*, 36–47.

35. Savva, M.; Chang, A.X.; Hanrahan, P.; Fisher, M.; Nießner, M. Pigraphs: Learning Interaction Snapshots from Observations. *ACM Trans. Graph.* **2016**, *35*, 1–12.

36. Shahroudy, A.; Liu, J.; Ng, T.T.; Wang, G. NTU RGB+D: A Large Scale Dataset for 3D Human Activity Analysis. *Proc. IEEE Comput. Soc. Conf. Comput. Vis. Pattern Recognit.* **2016**, *2016-Decem*, 1010–1019, doi:10.1109/CVPR.2016.115.

37. Gu, C.; Sun, C.; Ross, D.A.; Vondrick, C.; Pantofaru, C.; Li, Y.; Vijayanarasimhan, S.; Toderici, G.; Ricco, S.; Sukthankar, R.; et al. Ava: A Video Dataset of Spatio-Temporally Localized Atomic Visual Actions. In Proceedings of the Proceedings of the IEEE conference on computer vision and pattern recognition; 2018; pp. 6047–6056.

38. Amir, A.; Taba, B.; Berg, D.; Melano, T.; McKinstry, J.; Di Nolfo, C.; Nayak, T.; Andreopoulos, A.; Garreau, G.; Mendoza, M.; et al. A Low Power, Fully Event-Based Gesture Recognition System. In Proceedings of the Proceedings of the IEEE conference on computer vision and pattern recognition; 2017; pp. 7243–7252.

39. Jiang, Y.-G.; Wu, Z.; Wang, J.; Xue, X.; Chang, S.-F. Exploiting Feature and Class Relationships in Video Categorization with Regularized Deep Neural Networks. *IEEE Trans. Pattern Anal. Mach. Intell.* **2017**, *40*, 352–364.

40. Kay, W.; Carreira, J.; Simonyan, K.; Zhang, B.; Hillier, C.; Vijayanarasimhan, S.; Viola, F.; Green, T.; Back, T.; Natsev, P.; et al. The Kinetics Human Action Video Dataset. *arXiv Prepr. arXiv1705.06950* **2017**.

41. Kong, Y.; Fu, Y. Max-Margin Heterogeneous Information Machine for RGB-D Action Recognition. *Int. J. Comput. Vis.* **2017**, *123*, 350–371.

42. Liu, C.; Hu, Y.; Li, Y.; Song, S.; Liu, J. Pku-Mmd: A Large Scale Benchmark for Continuous Multi-Modal Human Action Understanding. *arXiv Prepr. arXiv1703.07475* **2017**.

43. Goyal, R.; Kahou, S.E.; Michalski, V.; Materzynska, J.; Westphal, S.; Kim, H.; Haenel, V.; Fruend, I.; Yianilos, P.; Mueller-Freitag, M.; et al. The "Something Something" Video Database for Learning and Evaluating Visual Common Sense. In Proceedings of the 2017 IEEE International Conference on Computer Vision (ICCV); IEEE, October 2017; pp. 5843–5851.

44. Mehta, D.; Rhodin, H.; Casas, D.; Fua, P.; Sotnychenko, O.; Xu, W.; Theobalt, C. Monocular 3d Human Pose

Estimation in the Wild Using Improved Cnn Supervision. In Proceedings of the 2017 international conference on 3D vision (3DV); 2017; pp. 506–516.

45. Damen, D.; Doughty, H.; Farinella, G.M.; Fidler, S.; Furnari, A.; Kazakos, E.; Moltisanti, D.; Munro, J.; Perrett, T.; Price, W.; et al. Scaling Egocentric Vision: The Epic-Kitchens Dataset. In Proceedings of the Proceedings of the European conference on computer vision (ECCV); 2018; pp. 720–736.

46. Carreira, J.; Noland, E.; Banki-Horvath, A.; Hillier, C.; Zisserman, A. A Short Note about Kinetics-600. *arXiv Prepr. arXiv1808.01340* **2018**.

47. Ji, Y.; Xu, F.; Yang, Y.; Shen, F.; Shen, H.T.; Zheng, W.-S. A Large-Scale RGB-D Database for Arbitrary-View Human Action Recognition. In Proceedings of the Proceedings of the 26th ACM international Conference on Multimedia; 2018; pp. 1510–1518.

48. Calabrese, E.; Taverni, G.; Awai Easthope, C.; Skriabine, S.; Corradi, F.; Longinotti, L.; Eng, K.; Delbruck, T. DHP19: Dynamic Vision Sensor 3D Human Pose Dataset. In Proceedings of the Proceedings of the IEEE/CVF conference on computer vision and pattern recognition workshops; 2019; p. 0.

49. Martin, M.; Roitberg, A.; Haurilet, M.; Horne, M.; Reiß, S.; Voit, M.; Stiefelhagen, R. Drive\&act: A Multi-Modal Dataset for Fine-Grained Driver Behavior Recognition in Autonomous Vehicles. In Proceedings of the Proceedings of the IEEE/CVF International Conference on Computer Vision; 2019; pp. 2801–2810.

50. Zhang, Y.; Cao, C.; Cheng, J.; Lu, H. Egogesture: A New Dataset and Benchmark for Egocentric Hand Gesture Recognition. *IEEE Trans. Multimed.* **2018**, *20*, 1038–1050.

51. Monfort, M.; Andonian, A.; Zhou, B.; Ramakrishnan, K.; Bargal, S.A.; Yan, T.; Brown, L.; Fan, Q.; Gutfreund, D.; Vondrick, C.; et al. Moments in Time Dataset: One Million Videos for Event Understanding. *IEEE Trans. Pattern Anal. Mach. Intell.* **2019**, *42*, 502–508.

52. Liu, J.; Shahroudy, A.; Perez, M.; Wang, G.; Duan, L.-Y.; Kot, A.C. NTU RGB+D 120: A Large-Scale Benchmark for 3D Human Activity Understanding. *IEEE Trans. Pattern Anal. Mach. Intell.* **2020**, *42*, 2684–2701, doi:10.1109/TPAMI.2019.2916873.

53. Hassan, M.; Choutas, V.; Tzionas, D.; Black, M.J. Resolving 3D Human Pose Ambiguities with 3D Scene Constraints. In Proceedings of the Proceedings of the IEEE/CVF international conference on computer vision; 2019; pp. 2282–2292.

54. Jang, J.; Kim, D.; Park, C.; Jang, M.; Lee, J.; Kim, J. ETRI-Activity3D: A Large-Scale RGB-D Dataset for Robots to Recognize Daily Activities of the Elderly. In Proceedings of the 2020 IEEE/RSJ International Conference on Intelligent Robots and Systems (IROS); 2020; pp. 10990–10997.

55. Wang, L.; Sun, B.; Robinson, J.; Jing, T.; Fu, Y. EV-Action: Electromyography-Vision Multi-Modal Action Dataset. In Proceedings of the 2020 15th IEEE International Conference on Automatic Face and Gesture Recognition (FG 2020); 2020; pp. 160–167.

56. Fieraru, M.; Zanfir, M.; Oneata, E.; Popa, A.-I.; Olaru, V.; Sminchisescu, C. Three-Dimensional Reconstruction of Human Interactions. In Proceedings of the Proceedings of the IEEE/CVF Conference on Computer Vision and Pattern Recognition; 2020; pp. 7214–7223.

57. Taheri, O.; Ghorbani, N.; Black, M.J.; Tzionas, D. GRAB: A Dataset of Whole-Body Human Grasping of Objects. In Proceedings of the Computer Vision--ECCV 2020: 16th European Conference, Glasgow, UK, August 23--28, 2020, Proceedings, Part IV 16; 2020; pp. 581–600.

58. Ben-Shabat, Y.; Yu, X.; Saleh, F.; Campbell, D.; Rodriguez-Opazo, C.; Li, H.; Gould, S. The Ikea Asm Dataset: Understanding People Assembling Furniture through Actions, Objects and Pose. In Proceedings of the Proceedings of the IEEE/CVF Winter Conference on Applications of Computer Vision; 2021; pp. 847–859.

59. Miech, A.; Alayrac, J.-B.; Laptev, I.; Sivic, J.; Zisserman, A. Rareact: A Video Dataset of Unusual Interactions. *arXiv Prepr. arXiv2008.01018* **2020**.

60. Hossen, M.A.; Hong, O.W.; Caesarendra, W. Investigation of the Unsupervised Machine Learning Techniques for Human Activity Discovery. In Proceedings of the Proceedings of the 2nd International Conference on Electronics, Biomedical Engineering, and Health Informatics; Triwiyanto, T., Rizal, A., Caesarendra, W., Eds.; Springer Nature Singapore: Singapore, 2022; pp. 499–514.

61. Ghorbani, S.; Mahdaviani, K.; Thaler, A.; Kording, K.; Cook, D.J.; Blohm, G.; Troje, N.F. MoVi: A Large Multi-Purpose Human Motion and Video Dataset. *PLoS One* **2021**, *16*, e0253157.

62. Li, T.; Liu, J.; Zhang, W.; Ni, Y.; Wang, W.; Li, Z. Uav-Human: A Large Benchmark for Human Behavior Understanding with Unmanned Aerial Vehicles. In Proceedings of the Proceedings of the IEEE/CVF conference on computer vision and pattern recognition; 2021; pp. 16266–16275.

63. Rai, N.; Chen, H.; Ji, J.; Desai, R.; Kozuka, K.; Ishizaka, S.; Adeli, E.; Niebles, J.C. Home Action Genome: Cooperative Compositional Action Understanding. In Proceedings of the Proceedings of the IEEE/CVF Conference on Computer Vision and Pattern Recognition; 2021; pp. 11184–11193.

64. Grauman, K.; Westbury, A.; Byrne, E.; Chavis, Z.; Furnari, A.; Girdhar, R.; Hamburger, J.; Jiang, H.; Liu, M.; Liu, X.; et al. Ego4d: Around the World in 3,000 Hours of Egocentric Video. In Proceedings of the Proceedings of the IEEE/CVF Conference on Computer Vision and Pattern Recognition; 2022; pp. 18995–19012.

65. Damen, D.; Doughty, H.; Farinella, G.M.; Furnari, A.; Kazakos, E.; Ma, J.; Moltisanti, D.; Munro, J.; Perrett, T.; Price, W.; et al. Rescaling Egocentric Vision: Collection, Pipeline and Challenges for Epic-Kitchens-100. *Int. J. Comput. Vis.* **2022**, 1–23.

66. Ellis, C.; Masood, S.Z.; Tappen, M.F.; LaViola, J.J.; Sukthankar, R. Exploring the Trade-off between Accuracy and Observational Latency in Action Recognition. *Int. J. Comput. Vis.* **2013**, *101*, 420–436.

67. Theodorakopoulos, I.; Kastaniotis, D.; Economou, G.; Fotopoulos, S. Pose-Based Human Action Recognition via Sparse Representation in Dissimilarity Space. *J. Vis. Commun. Image Represent.* **2014**, *25*, 12–23.

68. Kwapisz, J.R.; Weiss, G.M.; Moore, S.A. Activity Recognition Using Cell Phone Accelerometers. *ACM SigKDD Explor. Newsl.* **2011**, *12*, 74–82.

69. Dernbach, S.; Das, B.; Krishnan, N.C.; Thomas, B.L.; Cook, D.J. Simple and Complex Activity Recognition through Smart Phones. In Proceedings of the 2012 eighth international conference on intelligent environments; 2012; pp. 214–221.

70. Reiss, A.; Stricker, D. Introducing a New Benchmarked Dataset for Activity Monitoring. In Proceedings of the 2012 16th international symposium on wearable computers; 2012; pp. 108–109.

71. Zhang, M.; Sawchuk, A.A. USC-HAD: A Daily Activity Dataset for Ubiquitous Activity Recognition Using Wearable Sensors. In Proceedings of the Proceedings of the 2012 ACM conference on ubiquitous computing; 2012; pp. 1036–1043.

72. Chavarriaga, R.; Sagha, H.; Calatroni, A.; Digumarti, S.T.; Tröster, G.; Millán, J. del R.; Roggen, D. The Opportunity Challenge: A Benchmark Database for on-Body Sensor-Based Activity Recognition. *Pattern Recognit. Lett.* **2013**, *34*, 2033–2042.

73. Anguita, D.; Ghio, A.; Oneto, L.; Parra, X.; Reyes-Ortiz, J.L.; others A Public Domain Dataset for Human Activity Recognition Using Smartphones. In Proceedings of the Esann; 2013; Vol. 3, p. 3.

74. Micucci, D.; Mobilio, M.; Napoletano, P. Unimib Shar: A Dataset for Human Activity Recognition Using Acceleration Data from Smartphones. *Appl. Sci.* **2017**, *7*, 1101.

75. Bhat, G.; Tran, N.; Shill, H.; Ogras, U.Y. W-HAR: An Activity Recognition Dataset and Framework Using Low-Power Wearable Devices. *Sensors* **2020**, *20*, 5356.

76. Logacjov, A.; Bach, K.; Kongsvold, A.; Bårdstu, H.B.; Mork, P.J. HARTH: A Human Activity Recognition Dataset for Machine Learning. *Sensors* **2021**, *21*, 7853.

77. Gao, W.; Zhang, L.; Teng, Q.; He, J.; Wu, H. DanHAR: Dual Attention Network for Multimodal Human Activity Recognition Using Wearable Sensors. *Appl. Soft Comput.* **2021**, *111*, 107728.

78. Sikder, N.; Nahid, A.-A. KU-HAR: An Open Dataset for Heterogeneous Human Activity Recognition. *Pattern Recognit. Lett.* **2021**, *146*, 46–54.

79. Leotta, M.; Fasciglione, A.; Verri, A. Daily Living Activity Recognition Using Wearable Devices: A Features-Rich Dataset and a Novel Approach. In Proceedings of the Pattern Recognition. ICPR International Workshops and Challenges: Virtual Event, January 10--15, 2021, Proceedings, Part II; 2021; pp. 171–187.

80. HumanEva: Synchronized Video and Motion Capture Dataset for Evaluation of Articulated Human Motion Leonid Sigal and Michael J. Black. **2006**.

81. Lab, C.M.U.G. Carnegie Mellon University - CMU Graphics Lab - Motion Capture Library 2007.

82. Ionescu, C.; Papava, D.; Olaru, V.; Sminchisescu, C. Human3. 6m: Large Scale Datasets and Predictive Methods

for 3d Human Sensing in Natural Environments. *IEEE Trans. Pattern Anal. Mach. Intell.* **2013**, *36*, 1325–1339.

83. Von Marcard, T.; Henschel, R.; Black, M.J.; Rosenhahn, B.; Pons-Moll, G. Recovering Accurate 3d Human Pose in the Wild Using Imus and a Moving Camera. In Proceedings of the Proceedings of the European Conference on Computer Vision (ECCV); 2018; pp. 601–617.

84. Niemann, F.; Reining, C.; Moya Rueda, F.; Nair, N.R.; Steffens, J.A.; Fink, G.A.; Ten Hompel, M. Lara: Creating a Dataset for Human Activity Recognition in Logistics Using Semantic Attributes. *Sensors* **2020**, *20*, 4083.

85. Punnakkal, A.R.; Chandrasekaran, A.; Athanasiou, N.; Quiros-Ramirez, A.; Black, M.J. BABEL: Bodies, Action and Behavior with English Labels. In Proceedings of the Proceedings of the IEEE/CVF Conference on Computer Vision and Pattern Recognition; 2021; pp. 722–731.

86. Fieraru, M.; Zanfir, M.; Oneata, E.; Popa, A.-I.; Olaru, V.; Sminchisescu, C. Learning Complex 3D Human Self-Contact. In Proceedings of the Proceedings of the AAAI Conference on Artificial Intelligence; 2021; Vol. 35, pp. 1343–1351.

87. Fieraru, M.; Zanfir, M.; Pirlea, S.C.; Olaru, V.; Sminchisescu, C. Aifit: Automatic 3d Human-Interpretable Feedback Models for Fitness Training. In Proceedings of the Proceedings of the IEEE/CVF conference on computer vision and pattern recognition; 2021; pp. 9919–9928.

88. Zhang, H.; Ren, S.; Yuan, H.; Zhao, J.; Li, F.; Sun, S.; Liang, Z.; Yu, T.; Shen, Q.; Cao, X. MMVP: A Multimodal MoCap Dataset with Vision and Pressure Sensors. *arXiv Prepr. arXiv2403.17610* **2024**.

89. Ma, Y.; Zhou, G.; Wang, S.; Zhao, H.; Jung, W. SignFi: Sign Language Recognition Using WiFi. **2018**, *2*, doi:10.1145/3191755.

90. Hernangómez, R.; Santra, A.; Stańczak, S. Human Activity Classification with Frequency Modulated Continuous Wave Radar Using Deep Convolutional Neural Networks. In Proceedings of the 2019 International Radar Conference (RADAR); 2019; pp. 1–6.

91. Wang, F.; Feng, J.; Zhao, Y.; Zhang, X.; Zhang, S.; Han, J. Joint Activity Recognition and Indoor Localization with WiFi Fingerprints. *IEEE Access* **2019**, *7*, 80058–80068.

92. Fioranelli, D.F.; Shah, D.S.A.; Li1, H.; Shrestha, A.; Yang, D.S.; Kernec, D.J. Le Radar Sensing for Healthcare: Associate Editor Francesco Fioranelli on the Applications of Radar in Monitoring Vital Signs and Recognising Human Activity Patterns. *Electron. Lett.* **2019**, *55*, 1022–1024.

93. Zheng, Y.; Zhang, Y.; Qian, K.; Zhang, G.; Liu, Y.; Wu, C.; Yang, Z. Zero-Effort Cross-Domain Gesture Recognition with Wi-Fi. In Proceedings of the Proceedings of the 17th Annual International Conference on Mobile Systems, Applications, and Services; Association for Computing Machinery: New York, NY, USA, 2019; pp. 313–325.

94. Yousefi, S.; Narui, H.; Dayal, S.; Ermon, S.; Valaee, S. A Survey on Behavior Recognition Using WiFi Channel State Information. *IEEE Commum. Mag.* **2017**, *55*.

95. Chakraborty, M.; Kumawat, H.C.; Dhavale, S.V.; Bazil Raj, A.A. DIAT-MRadHAR: Radar Micro-Doppler Signature Dataset for Human Suspicious Activity Recognition 2022.

96. Yang, J.; Chen, X.; Zou, H.; Wang, D.; Xu, Q.; Xie, L. EfficientFi: Toward Large-Scale Lightweight WiFi Sensing via CSI Compression. *IEEE Internet Things J.* **2022**, *9*, 13086–13095, doi:10.1109/JIOT.2021.3139958.

97. Wang, D.; Yang, J.; Cui, W.; Xie, L.; Sun, S. CAUTION: A Robust WiFi-Based Human Authentication System via Few-Shot Open-Set Recognition. *IEEE Internet Things J.* **2022**, *9*, 17323–17333, doi:10.1109/JIOT.2022.3156099.

98. Ofli, F.; Chaudhry, R.; Kurillo, G.; Vidal, R.; Bajcsy, R. Berkeley Mhad: A Comprehensive Multimodal Human Action Database. In Proceedings of the 2013 IEEE workshop on applications of computer vision (WACV); 2013; pp. 53–60.

99. Zhang, W.; Liu, Z.; Zhou, L.; Leung, H.; Chan, A.B. Martial Arts, Dancing and Sports Dataset: A Challenging Stereo and Multi-View Dataset for 3d Human Pose Estimation. *Image Vis. Comput.* **2017**, *61*, 22–39.

100. Kong, Q.; Wu, Z.; Deng, Z.; Klinkigt, M.; Tong, B.; Murakami, T. Mmact: A Large-Scale Dataset for Cross Modal Human Action Understanding. In Proceedings of the Proceedings of the IEEE/CVF International Conference on Computer Vision; 2019; pp. 8658–8667.

101. Chapron, K.; Lapointe, P.; Bouchard, K.; Gaboury, S. Highly Accurate Bathroom Activity Recognition Using Infrared Proximity Sensors. *IEEE J. Biomed. Heal. Informatics* **2020**, *24*, 2368–2377, doi:10.1109/JBHI.2019.2963388.

102. Alam, M.A.U.; Mazzoni, F.; Rahman, M.M.; Widberg, J. LAMAR: LiDAR Based Multi-Inhabitant Activity Recognition.; Association for Computing Machinery: New York, NY, USA, 2021.

103. Bocus, M.J.; Li, W.; Vishwakarma, S.; Kou, R.; Tang, C.; Woodbridge, K.; Craddock, I.; McConville, R.; Santos-Rodriguez, R.; Chetty, K.; et al. OPERAnet, a Multimodal Activity Recognition Dataset Acquired from Radio Frequency and Vision-Based Sensors. *Sci. data* **2022**, *9*, 474.

104. Fan, L.; Wang, Z.; Wang, H. Human Activity Recognition Model Based on Decision Tree. In Proceedings of the 2013 International Conference on Advanced Cloud and Big Data; IEEE, December 2013; pp. 64–68.

105. Nurwulan, N.R.; Selamaj, G. Human Daily Activities Recognition Using Decision Tree. In Proceedings of the Journal of Physics: Conference Series; 2021; Vol. 1833, p. 12039.

106. Müller, P.N.; Rauterberg, F.; Achenbach, P.; Tregel, T.; Göbel, S. Physical Exercise Quality Assessment Using Wearable Sensors. In *Multimedia Tools and Applications*; Springer, 2021; Vol. 80, pp. 229–243.

107. Müller, P.N.; Rauterberg, F.; Achenbach, P.; Tregel, T.; Göbel, S. Physical Exercise Quality Assessment Using Wearable Sensors. In Proceedings of the Serious Games; Fletcher, B., Ma, M., Göbel, S., Baalsrud Hauge, J., Marsh, T., Eds.; Springer International Publishing: Cham, 2021; pp. 229–243.

108. Pärkkä, J.; Cluitmans, L.; Ermes, M. Personalization Algorithm for Real-Time Activity Recognition Using PDA, Wireless Motion Bands, and Binary Decision Tree. *IEEE Trans. Inf. Technol. Biomed.* **2010**, *14*, 1211–1215.

109. Balli, S.; Sa\ugba\cs, E.A.; Peker, M. Human Activity Recognition from Smart Watch Sensor Data Using a Hybrid of Principal Component Analysis and Random Forest Algorithm. *Meas. Control* **2019**, *52*, 37–45.

110. Dewi, C.; Chen, R.-C. Human Activity Recognition Based on Evolution of Features Selection and Random Forest. In Proceedings of the 2019 IEEE International Conference on Systems, Man and Cybernetics (SMC); IEEE, October 2019; pp. 2496–2501.

111. Zhu, Y.; Chen, W.; Guo, G. Evaluating Spatiotemporal Interest Point Features for Depth-Based Action Recognition. *Image Vis. Comput.* **2014**, *32*, 453–464.

112. Nunes, U.M.; Faria, D.R.; Peixoto, P. A Human Activity Recognition Framework Using Max-Min Features and Key Poses with Differential Evolution Random Forests Classifier. *Pattern Recognit. Lett.* **2017**, *99*, 21–31, doi:10.1016/j.patrec.2017.05.004.

113. Nurwulan, N.R.; Selamaj, G. Random Forest for Human Daily Activity Recognition. In Proceedings of the Journal of Physics: Conference Series; 2020; Vol. 1655, p. 12087.

114. Gan, L.; Chen, F. Human Action Recognition Using APJ3D and Random Forests. *J. Softw.* **2013**, *8*, doi:10.4304/jsw.8.9.2238-2245.

115. Cippitelli, E.; Gasparrini, S.; Gambi, E.; Spinsante, S. A Human Activity Recognition System Using Skeleton Data from RGBD Sensors. *Comput. Intell. Neurosci.* **2016**, *2016*, doi:10.1155/2016/4351435.

116. Tran, D.N.; Phan, D.D. Human Activities Recognition in Android Smartphone Using Support Vector Machine. In Proceedings of the 2016 7th international conference on intelligent systems, modelling and simulation (isms); 2016; pp. 64–68.

117. Anguita, D.; Ghio, A.; Oneto, L.; Parra, X.; Reyes-Ortiz, J.L. Human Activity Recognition on Smartphones Using a Multiclass Hardware-Friendly Support Vector Machine. In Proceedings of the Ambient Assisted Living and Home Care: 4th International Workshop, IWAAL 2012, Vitoria-Gasteiz, Spain, December 3-5, 2012. Proceedings 4; 2012; pp. 216–223.

118. Youngwook Kim; Hao Ling Human Activity Classification Based on Micro-Doppler Signatures Using a Support Vector Machine. *IEEE Trans. Geosci. Remote Sens.* **2009**, *47*, 1328–1337, doi:10.1109/TGRS.2009.2012849.

119. Shuvo, M.M.H.; Ahmed, N.; Nouduri, K.; Palaniappan, K. A Hybrid Approach for Human Activity Recognition with Support Vector Machine and 1D Convolutional Neural Network. In Proceedings of the 2020 IEEE Applied Imagery Pattern Recognition Workshop (AIPR); 2020; pp. 1–5.

120. Nurhanim, K.; Elamvazuthi, I.; Izhar, L.I.; Ganesan, T. Classification of Human Activity Based on Smartphone Inertial Sensor Using Support Vector Machine. In Proceedings of the 2017 ieee 3rd international symposium in robotics and manufacturing automation (roma); 2017; pp. 1–5.

121. Mohsen, S.; Elkaseer, A.; Scholz, S.G. Human Activity Recognition Using K-Nearest Neighbor Machine Learning Algorithm. In Proceedings of the Proceedings of the International Conference on Sustainable Design and

Manufacturing; 2021; pp. 304–313.

122. Attal, F.; Mohammed, S.; Dedabrishvili, M.; Chamroukhi, F.; Oukhellou, L.; Amirat, Y. Physical Human Activity Recognition Using Wearable Sensors. *Sensors* **2015**, *15*, 31314–31338, doi:10.3390/s151229858.

123. Kaghyan, S.; Sarukhanyan, H. Activity Recognition Using K-Nearest Neighbor Algorithm on Smartphone with Tri-Axial Accelerometer. *Int. J. Informatics Model. Anal. (IJIMA), ITHEA Int. Sci. Soc. Bulg.* **2012**, *1*, 146–156.

124. Al-Akam, R.; Paulus, D. RGBD Human Action Recognition Using Multi-Features Combination and k-Nearest Neighbors Classification. *Int. J. Adv. Comput. Sci. Appl.* **2017**, *8*, 383–389.

125. Mandong, A.; Munir, U. Smartphone Based Activity Recognition Using K-Nearest Neighbor Algorithm. In Proceedings of the Proceedings of the International Conference on Engineering Technologies, Konya, Turkey; 2018; pp. 26–28.

126. Madarshahian, R.; Caicedo, J.M. Human Activity Recognition Using Multinomial Logistic Regression. In Proceedings of the Model Validation and Uncertainty Quantification, Volume 3: Proceedings of the 33rd IMAC, A Conference and Exposition on Structural Dynamics, 2015; 2015; pp. 363–372.

127. Chamroukhi, F.; Mohammed, S.; Trabelsi, D.; Oukhellou, L.; Amirat, Y. Joint Segmentation of Multivariate Time Series with Hidden Process Regression for Human Activity Recognition. *Neurocomputing* **2013**, *120*, 633–644.

128. Zaki, Z.; Shah, M.A.; Wakil, K.; Sher, F. Logistic Regression Based Human Activities Recognition. *J. Mech. Contin. Math. Sci* **2020**, *15*, 228–246.

129. Minarno, A.E.; Kusuma, W.A.; Wibowo, H. Performance Comparisson Activity Recognition Using Logistic Regression and Support Vector Machine. In Proceedings of the 2020 3rd International conference on intelligent autonomous systems (ICoIAS); 2020; pp. 19–24.

130. Ambati, L.S.; El-Gayar, O. Human Activity Recognition: A Comparison of Machine Learning Approaches. *J. Midwest Assoc. Inf. Syst.* **2021**, *2021*, 4.

131. Sozinov, K.; Vlassov, V.; Girdzijauskas, S. Human Activity Recognition Using Federated Learning. In Proceedings of the 2018 IEEE Intl Conf on Parallel \& Distributed Processing with Applications, Ubiquitous Computing \& Communications, Big Data \& Cloud Computing, Social Computing \& Networking, Sustainable Computing \& Communications (ISPA/IUCC/BDCloud/SocialCom/SustainC; 2018; pp. 1103–1111.

132. Bustoni, I.A.; Hidayatulloh, I.; Ningtyas, A.M.; Purwaningsih, A.; Azhari, S.N. Classification Methods Performance on Human Activity Recognition. In Proceedings of the Journal of Physics: Conference Series; 2020; Vol. 1456, p. 12027.

133. Halim, N. Stochastic Recognition of Human Daily Activities via Hybrid Descriptors and Random Forest Using Wearable Sensors. *Array* **2022**, *15*, 100190.

134. Zeng, M.; Nguyen, L.T.; Yu, B.; Mengshoel, O.J.; Zhu, J.; Wu, P.; Zhang, J. Convolutional Neural Networks for Human Activity Recognition Using Mobile Sensors. In Proceedings of the 6th international conference on mobile

computing, applications and services; 2014; pp. 197–205.

135. Wang, L. Recognition of Human Activities Using Continuous Autoencoders with Wearable Sensors. *Sensors* **2016**, *16*, 189.

136. Shen, J.; Fang, H. Human Activity Recognition Using Gaussian Naive Bayes Algorithm in Smart Home. In Proceedings of the Journal of Physics: Conference Series; 2020; Vol. 1631, p. 12059.

137. Kose, M.; Incel, O.D.; Ersoy, C. Online Human Activity Recognition on Smart Phones. In Proceedings of the Workshop on mobile sensing: from smartphones and wearables to big data; 2012; Vol. 16, pp. 11–15.

138. Sarkar, A.M.J.; Lee, Y.-K.; Lee, S. A Smoothed Naive Bayes-Based Classifier for Activity Recognition. *IETE Tech. Rev.* **2010**, *27*, 107–119.

139. Zhang, W.; Zhao, X.; Li, Z. A Comprehensive Study of Smartphone-Based Indoor Activity Recognition via Xgboost. *IEEE Access* **2019**, *7*, 80027–80042.

140. Bukht, T.F.N.; Jalal, A. A Robust Model of Human Activity Recognition Using Independent Component Analysis and XGBoost. In Proceedings of the 2024 5th International Conference on Advancements in Computational Sciences (ICACS); 2024; pp. 1–7.

141. O'Halloran, J.; Curry, E. A Comparison of Deep Learning Models in Human Activity Recognition and Behavioural Prediction on the MHEALTH Dataset. In Proceedings of the AICS; 2019; pp. 212–223.

142. Ayumi, V. Pose-Based Human Action Recognition with Extreme Gradient Boosting. In Proceedings of the 2016 IEEE student conference on research and development (SCOReD); 2016; pp. 1–5.

143. Zhang, L.; Wu, X.; Luo, D. Human Activity Recognition with HMM-DNN Model. In Proceedings of the 2015 IEEE 14th International Conference on Cognitive Informatics \& Cognitive Computing (ICCI* CC); 2015; pp. 192–197.

144. Ronao, C.A.; Cho, S.-B. Human Activity Recognition Using Smartphone Sensors with Two-Stage Continuous Hidden Markov Models. In Proceedings of the 2014 10th international conference on natural computation (ICNC); 2014; pp. 681–686.

145. San-Segundo, R.; Montero, J.M.; Moreno-Pimentel, J.; Pardo, J.M. HMM Adaptation for Improving a Human Activity Recognition System. *Algorithms* **2016**, *9*, 60.

146. Xia, L.; Chen, C.-C.; Aggarwal, J.K. View Invariant Human Action Recognition Using Histograms of 3d Joints. In Proceedings of the 2012 IEEE computer society conference on computer vision and pattern recognition workshops; 2012; pp. 20–27.

147. Kwon, Y.; Kang, K.; Bae, C. Unsupervised Learning for Human Activity Recognition Using Smartphone Sensors. *Expert Syst. Appl.* **2014**, *41*, 6067–6074, doi:10.1016/j.eswa.2014.04.037.

148. Jamel, A.A.M.; Akay, B. Human Activity Recognition Based on Parallel Approximation Kernel K-Means

Algorithm. *Comput. Syst. Sci. \& Eng.* **2020**, *35*.

149. Manzi, A.; Dario, P.; Cavallo, F. A Human Activity Recognition System Based on Dynamic Clustering of Skeleton Data. *Sensors (Switzerland)* **2017**, *17*, doi:10.3390/s17051100.

150. Colpas, P.A.; Vicario, E.; De-La-Hoz-Franco, E.; Pineres-Melo, M.; Oviedo-Carrascal, A.; Patara, F. Unsupervised Human Activity Recognition Using the Clustering Approach: A Review. *Sensors (Switzerland)* **2020**, *20*, doi:10.3390/s20092702.

151. Gaglio, S.; Re, G. Lo; Morana, M. Human Activity Recognition Process Using 3-D Posture Data. *IEEE Trans. Human-Machine Syst.* **2015**, *45*, 586[1] S. Gaglio, G. Lo Re, and M. Morana, "Human, doi:10.1109/THMS.2014.2377111.

152. Machado, I.P.; Gomes, A.L.; Gamboa, H.; Paixão, V.; Costa, R.M. Human Activity Data Discovery from Triaxial Accelerometer Sensor: Non-Supervised Learning Sensitivity to Feature Extraction Parametrization. *Inf. Process. \& Manag.* **2015**, *51*, 204–214.

153. Wang, T.; Ng, W.W.Y.; Li, J.; Wu, Q.; Zhang, S.; Nugent, C.; Shewell, C. A Deep Clustering via Automatic Feature Embedded Learning for Human Activity Recognition. *IEEE Trans. Circuits Syst. Video Technol.* **2021**, *32*, 210–223.

154. Liu, A.-A.; Su, Y.-T.; Nie, W.-Z.; Kankanhalli, M. Hierarchical Clustering Multi-Task Learning for Joint Human Action Grouping and Recognition. *IEEE Trans. Pattern Anal. Mach. Intell.* **2016**, *39*, 102–114.

155. Ouyang, X.; Xie, Z.; Zhou, J.; Xing, G.; Huang, J. ClusterFL: A Clustering-Based Federated Learning System for Human Activity Recognition. *ACM Trans. Sens. Networks* **2022**, *19*, 1–32.

156. Mejia-Ricart, L.F.; Helling, P.; Olmsted, A. Evaluate Action Primitives for Human Activity Recognition Using Unsupervised Learning Approach. In Proceedings of the 2017 12th International conference for internet technology and secured transactions (ICITST); 2017; pp. 186–188.

157. Dobbins, C.; Rawassizadeh, R. Towards Clustering of Mobile and Smartwatch Accelerometer Data for Physical Activity Recognition. In Proceedings of the Informatics; 2018; Vol. 5, p. 29.

158. Fahad, L.G.; Tahir, S.F.; Rajarajan, M. Activity Recognition in Smart Homes Using Clustering Based Classification. In Proceedings of the 2014 22nd International conference on pattern recognition; 2014; pp. 1348–1353.

159. Piyathilaka, L.; Kodagoda, S. Gaussian Mixture Based HMM for Human Daily Activity Recognition Using 3D Skeleton Features. In Proceedings of the 2013 IEEE 8th conference on industrial electronics and applications (ICIEA); 2013; pp. 567–572.

160. Cheng, X.; Huang, B. CSI-Based Human Continuous Activity Recognition Using GMM--HMM. *IEEE Sens. J.* **2022**, *22*, 18709–18717.

161. Srivastava, P.; Wong, W.C. Hierarchical Human Activity Recognition Using GMM. In Proceedings of the AMBIENT 2012: The Second International Conference on Ambient Computing, Applications, Services and

Technologies; 2012; pp. 32–37.

162. Yang, X.; others Personalized Semi-Supervised Federated Learning for Human Activity Recognition. *arXiv Prepr. arXiv2104.08094* **2021**.

163. Vavoulas, G.; Chatzaki, C.; Malliotakis, T.; Pediaditis, M.; Tsiknakis, M. The MobiAct Dataset: Recognition of Activities of Daily Living Using Smartphones. *ICT4AWE 2016 - 2nd Int. Conf. Inf. Commun. Technol. Ageing Well e-Health, Proc.* **2016**, 143–151, doi:10.5220/0005792401430151.

164. Kwapisz, J.R.; Weiss, G.M.; Moore, S.A. Activity Recognition Using Cell Phone Accelerometers. *ACM SIGKDD Explor. Newsl.* **2011**, *12*, 74–82, doi:10.1145/1964897.1964918.

165. Yang, S.-H.; Baek, D.-G.; Thapa, K. Semi-Supervised Adversarial Learning Using LSTM for Human Activity Recognition. *Sensors* **2022**, *22*, 4755.

166. van Kasteren, T.L.M.; Englebienne, G.; Kröse, B.J.A. Human Activity Recognition from Wireless Sensor Network Data: Benchmark and Software. In *Activity recognition in pervasive intelligent environments*; Springer, 2011; pp. 165–186.

167. Cook, D.J.; Schmitter-Edgecombe, M. Assessing the Quality of Activities in a Smart Environment. *Methods Inf. Med.* **2009**, *48*, 480–485.

168. Oh, S.; Ashiquzzaman, A.; Lee, D.; Kim, Y.; Kim, J. Study on Human Activity Recognition Using Semi-Supervised Active Transfer Learning. *Sensors* **2021**, *21*, 2760.

169. Qu, Y.; Tang, Y.; Yang, X.; Wen, Y.; Zhang, W. Context-Aware Mutual Learning for Semi-Supervised Human Activity Recognition Using Wearable Sensors. *Expert Syst. Appl.* **2023**, *219*, 119679.

170. Wan, S.; Qi, L.; Xu, X.; Tong, C.; Gu, Z. Deep Learning Models for Real-Time Human Activity Recognition with Smartphones. *Mob. Networks Appl.* **2020**, *25*, 743–755, doi:10.1007/s11036-019-01445-x.

171. Banos, O.; Garcia, R.; Holgado-Terriza, J.A.; Damas, M.; Pomares, H.; Rojas, I.; Saez, A.; Villalonga, C. MHealthDroid: A Novel Framework for Agile Development of Mobile Health Applications. In Proceedings of the Ambient Assisted Living and Daily Activities; Pecchia, L., Chen, L.L., Nugent, C., Bravo, J., Eds.; Springer International Publishing: Cham, 2014; pp. 91–98.

172. Kim, D.; Han, S.; Son, H.; Lee, D. Human Activity Recognition Using Semi-Supervised Multi-Modal DEC for Instagram Data. In; 2020; pp. 869–880 ISBN 978-3-030-47425-6.

173. Bi, H.; Perello-Nieto, M.; Santos-Rodriguez, R.; Flach, P.; Craddock, I. An Active Semi-Supervised Deep Learning Model for Human Activity Recognition. *J. Ambient Intell. Humaniz. Comput.* **2023**, *14*, 13049–13065.

174. Paola Patricia, A.C.; Rosberg, P.C.; Butt-Aziz, S.; Marlon Alberto, P.M.; Roberto-Cesar, M.O.; Miguel, U.T.; Naz, S. Semi-Supervised Ensemble Learning for Human Activity Recognition in Casas Kyoto Dataset. *Heliyon* **2024**, *10*, doi:10.1016/j.heliyon.2024.e29398.

175. Presotto, R.; Civitarese, G.; Bettini, C. Federated Clustering and Semi-Supervised Learning: A New Partnership for Personalized Human Activity Recognition. *Pervasive Mob. Comput.* **2023**, *88*, 101726.

176. Zeng, M.; Yu, T.; Wang, X.; Nguyen, L.T.; Mengshoel, O.J.; Lane, I. Semi-Supervised Convolutional Neural Networks for Human Activity Recognition. In Proceedings of the 2017 IEEE International Conference on Big Data (Big Data); 2017; pp. 522–529.

177. Zilelioglu, H.; Khodabandelou, G.; Chibani, A.; Amirat, Y. Semisupervised Generative Adversarial Networks With Temporal Convolutions for Human Activity Recognition. *IEEE Sens. J.* **2023**, *23*, 12355–12369, doi:10.1109/JSEN.2023.3267243.

178. Wang, P.; Li, W.; Gao, Z.; Zhang, Y.; Tang, C.; Ogunbona, P. Scene Flow to Action Map: A New Representation for Rgb-d Based Action Recognition with Convolutional Neural Networks. In Proceedings of the CVPR; 2017.

179. Wan, J.; Zhao, Y.; Zhou, S.; Guyon, I.; Escalera, S.; Li, S.Z. Chalearn Looking at People Rgb-d Isolated and Continuous Datasets for Gesture Recognition. In Proceedings of the Proceedings of the IEEE conference on computer vision and pattern recognition workshops; 2016; pp. 56–64.

180. Liu, A.-A.; Xu, N.; Nie, W.-Z.; Su, Y.-T.; Wong, Y.; Kankanhalli, M. Benchmarking a Multimodal and Multiview and Interactive Dataset for Human Action Recognition. *IEEE Trans. Cybern.* **2016**, *47*, 1781–1794.

181. Ke, Q.; An, S.; Bennamoun, M.; Sohel, F.; Boussaid, F. Skeletonnet: Mining Deep Part Features for 3-d Action Recognition. *IEEE signal Process. lett.* **2017**, *24*.

182. Rahmani, H.; Bennamoun, M. Learning Action Recognition Model from Depth and Skeleton Videos. In Proceedings of the ICCV; 2017.

183. Yan, S.; Xiong, Y.; Lin, D. Spatial Temporal Graph Convolutional Networks for Skeleton-Based Action Recognition. In Proceedings of the Proceedings of the Thirty-Second AAAI Conference on Artificial Intelligence and Thirtieth Innovative Applications of Artificial Intelligence Conference and Eighth AAAI Symposium on Educational Advances in Artificial Intelligence; AAAI Press, 2018.

184. Wang, L.; Xiong, Y.; Wang, Z.; Qiao, Y.; Lin, D.; Tang, X.; Van Gool, L. Temporal Segment Networks for Action Recognition in Videos. *IEEE Trans. Pattern Anal. Mach. Intell.* **2018**, *41*, 2740–2755.

185. Li, M.; Chen, S.; Chen, X.; Zhang, Y.; Wang, Y.; Tian, Q. Actional-Structural Graph Convolutional Networks for Skeleton-Based Action Recognition. In Proceedings of the 2019 IEEE/CVF Conference on Computer Vision and Pattern Recognition (CVPR); IEEE, June 2019; pp. 3590–3598.

186. Song, Y.-F.; Zhang, Z.; Shan, C.; Wang, L. Richly Activated Graph Convolutional Network for Robust Skeleton-Based Action Recognition. *IEEE Trans. Circuits Syst. Video Technol.* **2021**, *31*, 1915–1925, doi:10.1109/TCSVT.2020.3015051.

187. Shi, L.; Zhang, Y.; Cheng, J.; Lu, H. Two-Stream Adaptive Graph Convolutional Networks for Skeleton-Based Action Recognition. In Proceedings of the 2019 IEEE/CVF Conference on Computer Vision and Pattern

Recognition (CVPR); IEEE, June 2019; pp. 12018–12027.

188. Shi, L.; Zhang, Y.; Cheng, J.; Lu, H. Skeleton-Based Action Recognition With Directed Graph Neural Networks. In Proceedings of the 2019 IEEE/CVF Conference on Computer Vision and Pattern Recognition (CVPR); IEEE, June 2019; pp. 7904–7913.

189. Yang, H.; Yan, D.; Zhang, L.; Sun, Y.; Li, D.; Maybank, S.J. Feedback Graph Convolutional Network for Skeleton-Based Action Recognition. *IEEE Trans. Image Process.* **2022**, *31*, 164–175, doi:10.1109/TIP.2021.3129117.

190. Cheng, K.; Zhang, Y.; He, X.; Chen, W.; Cheng, J.; Lu, H. Skeleton-Based Action Recognition With Shift Graph Convolutional Network. In Proceedings of the 2020 IEEE/CVF Conference on Computer Vision and Pattern Recognition (CVPR); IEEE, June 2020; pp. 180–189.

191. Shi, L.; Zhang, Y.; Cheng, J.; Lu, H. Decoupled Spatial-Temporal Attention Network for Skeleton-Based Action-Gesture Recognition. In *Proceedings of the Asian conference on computer vision*; 2021; pp. 38–53.

192. Liu, Z.; Zhang, H.; Chen, Z.; Wang, Z.; Ouyang, W. Disentangling and Unifying Graph Convolutions for Skeleton-Based Action Recognition. In Proceedings of the Proceedings of the IEEE/CVF conference on computer vision and pattern recognition; 2020; pp. 143–152.

193. Kwon, H.; Kim, M.; Kwak, S.; Cho, M. Learning Self-Similarity in Space and Time as Generalized Motion for Video Action Recognition. In Proceedings of the 2021 IEEE/CVF International Conference on Computer Vision (ICCV); IEEE, October 2021; pp. 13045–13055.

194. Davoodikakhki, M.; Yin, K. Hierarchical Action Classification with Network Pruning. In *Advances in Visual Computing: 15th International Symposium, ISVC 2020, San Diego, CA, USA, October 5--7, 2020, Proceedings, Part I 15*; 2020; pp. 291–305.

195. Das, S.; Dai, R.; Yang, D.; Bremond, F. Vpn++: Rethinking Video-Pose Embeddings for Understanding Activities of Daily Living. *IEEE Trans. Pattern Anal. Mach. Intell.* **2021**, *44*, 9703–9717.

196. Liu, Z.; Ning, J.; Cao, Y.; Wei, Y.; Zhang, Z.; Lin, S.; Hu, H. Video Swin Transformer. In Proceedings of the 2022 IEEE/CVF Conference on Computer Vision and Pattern Recognition (CVPR); IEEE, June 2022; pp. 3192–3201.

197. Duan, H.; Zhao, Y.; Xiong, Y.; Liu, W.; Lin, D. Omni-Sourced Webly-Supervised Learning for Video Recognition. In *European conference on computer vision*; 2020; pp. 670–688.

198. Duan, H.; Zhao, Y.; Chen, K.; Lin, D.; Dai, B. Revisiting Skeleton-Based Action Recognition. In Proceedings of the Proceedings of the IEEE/CVF Conference on Computer Vision and Pattern Recognition; 2022; pp. 2969–2978.

199. Carreira, J.; Zisserman, A. Quo Vadis, Action Recognition? A New Model and the Kinetics Dataset. In Proceedings of the CVPR; 2017.

200. Girdhar, R.; Joao Carreira, J.; Doersch, C.; Zisserman, A. Video Action Transformer Network. In Proceedings of the 2019 IEEE/CVF Conference on Computer Vision and Pattern Recognition (CVPR); IEEE, June 2019; pp. 244–253.

201. Li, S.; Cao, Q.; Liu, L.; Yang, K.; Liu, S.; Hou, J.; Yi, S. GroupFormer: Group Activity Recognition with Clustered Spatial-Temporal Transformer. In Proceedings of the ICCV; 2021.

202. Neimark, D.; Bar, O.; Zohar, M.; Asselmann, D. Video Transformer Network. In Proceedings of the ICCV; 2021.

203. Zhang, Y.; Li, X.; Liu, C.; Shuai, B.; Zhu, Y.; Brattoli, B.; Chen, H.; Marsic, I.; Tighe, J. Vidtr: Video Transformer without Convolutions. In Proceedings of the ICCV; 2021.

204. Bertasius, G.; Wang, H.; Torresani, L. Is Space-Time Attention All You Need for Video Understanding? In Proceedings of the ICML; 2021.

205. Bulat, A.; Perez Rua, J.M.; Sudhakaran, S.; Martinez, B.; Tzimiropoulos, G. Space-Time Mixing Attention for Video Transformer. *NeurIPS* **2021**.

206. Patrick, M.; Campbell, D.; Asano, Y.; Misra, I.; Metze, F.; Feichtenhofer, C.; Vedaldi, A.; Henriques, J.F. Keeping Your Eye on the Ball: Trajectory Attention in Video Transformers. *NeurIPS* **2021**.

207. Fan, H.; Xiong, B.; Mangalam, K.; Li, Y.; Yan, Z.; Malik, J.; Feichtenhofer, C. Multiscale Vision Transformers. In Proceedings of the ICCV; 2021.

208. Arnab, A.; Dehghani, M.; Heigold, G.; Sun, C.; Lučić, M.; Schmid, C. Vivit: A Video Vision Transformer. In Proceedings of the ICCV; 2021.

209. Ryoo, M.S.; Piergiovanni, A.J.; Arnab, A.; Dehghani, M.; Angelova, A. TokenLearner: What Can 8 Learned Tokens Do for Images and Videos? *arXiv Prepr. arXiv2106.11297* **2021**.

210. Zha, X.; Zhu, W.; Xun, L.T.; Yang, S.; Liu, J. Shifted Chunk Transformer for Spatio-Temporal Representational Learning. In Proceedings of the NeurIPS; 2021.

211. Zhang, F.Z.; Campbell, D.; Gould, S. Efficient Two-Stage Detection of Human-Object Interactions with a Novel Unary-Pairwise Transformer. In Proceedings of the CVPR; 2022.

212. Chao, Y.-W.; Liu, Y.; Liu, X.; Zeng, H.; Deng, J. Learning to Detect Human-Object Interactions. *Proc. - 2018 IEEE Winter Conf. Appl. Comput. Vision, WACV 2018* **2017**, *2018-Janua*, 381–389, doi:10.1109/WACV.2018.00048.

213. Gupta, S.; Malik, J. Visual Semantic Role Labeling. **2015**.

214. Guo, H.; Wang, H.; Ji, Q. Uncertainty-Guided Probabilistic Transformer for Complex Action Recognition. In Proceedings of the CVPR; 2022.

215. Kuehne, H.; Arslan, A.; Serre, T. The Language of Actions: Recovering the Syntax and Semantics of Goal-Directed Human Activities. In Proceedings of the 2014 IEEE Conference on Computer Vision and Pattern Recognition; IEEE, June 2014; pp. 780–787.

216. Idrees, H.; Zamir, A.R.; Jiang, Y.G.; Gorban, A.; Laptev, I.; Sukthankar, R.; Shah, M. The THUMOS Challenge on Action Recognition for Videos "in the Wild." *Comput. Vis. Image Underst.* **2017**, *155*, 1–23,

doi:10.1016/j.cviu.2016.10.018.

217. Chen, C.-F.; Panda, R.; Fan, Q. Regionvit: Regional-to-Local Attention for Vision Transformers. In Proceedings of the ICLR; 2022.

218. Yang, J.; Dong, X.; Liu, L.; Zhang, C.; Shen, J.; Yu, D. Recurring the Transformer for Video Action Recognition. In Proceedings of the CVPR; 2022.

219. Materzynska, J.; Berger, G.; Bax, I.; Memisevic, R. The Jester Dataset: A Large-Scale Video Dataset of Human Gestures. In Proceedings of the Proceedings of the IEEE/CVF international conference on computer vision workshops; 2019; p. 0.

220. Truong, T.-D.; Bui, Q.-H.; Duong, C.N.; Seo, H.-S.; Phung, S.L.; Li, X.; Luu, K. Direcformer: A Directed Attention in Transformer Approach to Robust Action Recognition. In Proceedings of the CVPR; 2022.

221. Li, K.; Wang, Y.; Gao, P.; Song, G.; Liu, Y.; Li, H.; Qiao, Y. Uniformer: Unified Transformer for Efficient Spatiotemporal Representation Learning. In Proceedings of the ICLR; 2022.

222. Fan, Q.; Chen, C.-F.; Panda, R. Can an Image Classifier Suffice For Action Recognition? In Proceedings of the ICLR; 2022.

223. Yan, S.; Xiong, X.; Arnab, A.; Lu, Z.; Zhang, M.; Sun, C.; Schmid, C. Multiview Transformers for Video Recognition. *CVPR* **2022**.

224. Ranasinghe, K.; Naseer, M.; Khan, S.; Khan, F.S.; Ryoo, M.S. Self-Supervised Video Transformer. In Proceedings of the CVPR; 2022.

225. Wang, R.; Chen, D.; Wu, Z.; Chen, Y.; Dai, X.; Liu, M.; Jiang, Y.-G.; Zhou, L.; Yuan, L. Bevt: Bert Pretraining of Video Transformers. In Proceedings of the Proceedings of the IEEE/CVF conference on computer vision and pattern recognition; 2022; pp. 14733–14743.

226. Tong, Z.; Song, Y.; Wang, J.; Wang, L. Videomae: Masked Autoencoders Are Data-Efficient Learners for Self-Supervised Video Pre-Training. *Adv. Neural Inf. Process. Syst.* **2022**, *35*, 10078–10093.

227. Feichtenhofer, C.; Li, Y.; He, K.; others Masked Autoencoders as Spatiotemporal Learners. *Adv. Neural Inf. Process. Syst.* **2022**, *35*, 35946–35958.

228. Wang, J.; Bertasius, G.; Tran, D.; Torresani, L. Long-Short Temporal Contrastive Learning of Video Transformers. In Proceedings of the Proceedings of the IEEE/CVF Conference on Computer Vision and Pattern Recognition; 2022; pp. 14010–14020.

229. Girdhar, R.; El-Nouby, A.; Singh, M.; Alwala, K.V.; Joulin, A.; Misra, I. Omnimae: Single Model Masked Pretraining on Images and Videos. In Proceedings of the Proceedings of the IEEE/CVF conference on computer vision and pattern recognition; 2023; pp. 10406–10417.

230. Russakovsky, O.; Deng, J.; Su, H.; Krause, J.; Satheesh, S.; Ma, S.; Huang, Z.; Karpathy, A.; Khosla, A.; Bernstein,

M.; et al. ImageNet Large Scale Visual Recognition Challenge. *Int. J. Comput. Vis.* **2014**, *115*, 211–252, doi:10.1007/s11263-015-0816-y.

231. Sun, X.; Chen, P.; Chen, L.; Li, C.; Li, T.H.; Tan, M.; Gan, C. Masked Motion Encoding for Self-Supervised Video Representation Learning. In Proceedings of the Proceedings of the IEEE/CVF Conference on Computer Vision and Pattern Recognition; 2023; pp. 2235–2245.

232. Fan, D.; Wang, J.; Liao, S.; Zhu, Y.; Bhat, V.; Santos-Villalobos, H.; MV, R.; Li, X. Motion-Guided Masking for Spatiotemporal Representation Learning. In Proceedings of the Proceedings of the IEEE/CVF International Conference on Computer Vision; 2023; pp. 5619–5629.

233. Xu, W.; Yu, J.; Miao, Z.; Wan, L.; Ji, Q. Spatio-Temporal Deep Q-Networks for Human Activity Localization. *IEEE Trans. Circuits Syst. Video Technol.* **2019**, *30*, 2984–2999.

234. Tang, Y.; Tian, Y.; Lu, J.; Li, P.; Zhou, J. Deep Progressive Reinforcement Learning for Skeleton-Based Action Recognition. In Proceedings of the CVPR; 2018.

235. Wu, W.; He, D.; Tan, X.; Chen, S.; Wen, S. Multi-Agent Reinforcement Learning Based Frame Sampling for Effective Untrimmed Video Recognition. In Proceedings of the Proceedings of the IEEE/CVF International Conference on Computer Vision; 2019; pp. 6222–6231.

236. Lu, Y.; Li, Y.; Velipasalar, S. Efficient Human Activity Classification from Egocentric Videos Incorporating Actor-Critic Reinforcement Learning. In Proceedings of the 2019 IEEE International Conference on Image Processing (ICIP); 2019; pp. 564–568.

237. Kumrai, T.; Korpela, J.; Maekawa, T.; Yu, Y.; Kanai, R. Human Activity Recognition with Deep Reinforcement Learning Using the Camera of a Mobile Robot. In Proceedings of the 2020 IEEE international conference on pervasive computing and communications (PerCom); 2020; pp. 1–10.

238. Ma, Y.; Arshad, S.; Muniraju, S.; Torkildson, E.; Rantala, E.; Doppler, K.; Zhou, G. Location-and Person-Independent Activity Recognition with WiFi, Deep Neural Networks, and Reinforcement Learning. *ACM Trans. Internet Things* **2021**, *2*, 1–25.

239. Palipana, S.; Rojas, D.; Agrawal, P.; Pesch, D. FallDeFi: Ubiquitous Fall Detection Using Commodity Wi-Fi Devices. *Proc. ACM Interactive, Mobile, Wearable Ubiquitous Technol.* **2018**, *1*, 1–25.

240. Gowda, S.N.; Sevilla-Lara, L.; Keller, F.; Rohrbach, M. Claster: Clustering with Reinforcement Learning for Zero-Shot Action Recognition. In Proceedings of the European Conference on Computer Vision; 2022; pp. 187–203.

Disclaimer/Publisher's Note: The statements, opinions and data contained in all publications are solely those of the individual author(s) and contributor(s) and not of MDPI and/or the editor(s). MDPI and/or the editor(s) disclaim responsibility for any injury to people or property resulting from any ideas, methods, instructions or products referred to in the content.
